# Borderline ovarian tumor frozen section diagnoses with features suspicious of invasive cancer: a retrospective study

**DOI:** 10.1186/s13048-021-00897-8

**Published:** 2021-10-22

**Authors:** Koen De Decker, Karina H. Jaroch, Joost Bart, Loes F. S. Kooreman, Roy F. P. M. Kruitwagen, Hans W. Nijman, Arnold-Jan Kruse

**Affiliations:** 1 Department of Obstetrics and Gynaecology, Isala Hospital, Zwolle, The Netherlands; 2 Department of Obstetrics and Gynaecology, University Medical Center Groningen, PO Box 30.001, 9700 RB Groningen, The Netherlands; 3 University of Groningen, Groningen, The Netherlands; 4 Department Pathology and Medical Biology, University of Groningen, University Medical Center Groningen, Groningen, The Netherlands; 5 Department of Pathology, Maastricht University Medical Centre, Maastricht, The Netherlands; 6 Department of Obstetrics and Gynaecology, Maastricht University Medical Centre, Maastricht, The Netherlands; 7 GROW, School for Oncology and Developmental Biology, Maastricht University Medical Centre, Maastricht, The Netherlands

**Keywords:** Borderline tumors of the ovary, Frozen section, Ovarian cancer, Ovarian neoplasm, Operative surgical procedure

## Abstract

**Background:**

A frozen section diagnosis of a borderline ovarian tumor with suspicious features of invasive carcinoma (“at least borderline” or synonymous descriptions) presents us with the dilemma of whether or not to perform a full ovarian cancer staging procedure. Quantification of this dilemma may help us with the issue of this clinical decision. The present study assessed and compared both the prevalence of straightforward borderline and “at least borderline” frozen section diagnoses and the proportion of these women with a final histopathological diagnosis of invasive carcinoma, with a special interest in histologic subtypes.

**Methods:**

A retrospective cohort study was performed in three hospitals in The Netherlands. All women that underwent ovarian surgery with perioperative frozen section evaluation in one of these hospitals between January 2007 and July 2018 were identified and included in case of a borderline or “at least borderline” frozen section diagnosis and a borderline ovarian tumor or invasive carcinoma as a final diagnosis.

**Results:**

A total of 223 women were included, of which 41 women (18.4%) were diagnosed with “at least borderline” at frozen section. Thirteen of forty-one women (31.7%) following “at least borderline” frozen section diagnosis and 14 of 182 women (7.7%) following a straightforward borderline frozen section diagnosis were diagnosed with invasive carcinoma at paraffin section evaluation (*p* < 0.001). When compared to straightforward borderline frozen section diagnoses, the proportion of women diagnosed with invasive carcinoma increased from 3.1 to 35.7% for serous tumors (*p* = 0.001), 10.0 to 21.7% for mucinous tumors (*p* = 0.129) and 50.0 to 75.0% (*p* = 0.452) in case of other histologic subtypes following an “at least borderline” frozen section diagnosis.

**Conclusions:**

Overall, when compared to women with a decisive borderline frozen section diagnosis, women diagnosed with “at least borderline” frozen section diagnoses were found to have a higher chance of carcinoma upon final diagnosis (7.7% vs 31.7%). Especially in the serous subtype, full staging during initial surgery might be considered after preoperative consent to prevent a second surgical procedure or chemotherapy in unstaged women. Further studies are needed to evaluate whether additional sampling in case of an “at least borderline” diagnosis may decrease the risk of surgical over-treatment.

## Background

Borderline ovarian tumors (synonymous with “atypical proliferative tumor”) are composed of mild to moderately atypical epithelial cells that show proliferation greater than that seen in benign tumors, but less than their malignant counterparts. Although usually absent in borderline ovarian tumors, one or more foci of stromal invasion of < 5 mm in the largest linear area might be present and should be classified and treated as borderline ovarian tumor. Mucinous borderline tumors account for approximately 40% of all borderline tumors and serous borderline tumors for approximately 50% [1, 2]. Adequate staging in case of borderline ovarian tumor diagnosis includes careful inspection of the peritoneum, peritoneal washing, (at least infracolic) omentectomy and peritoneal staging biopsies (pelvic peritoneum, paracolic gutters, diaphragm (4–6 biopsies)) [3]. In women with clinical early-stage ovarian cancer a full surgical staging should be performed, which means that pelvic and para-aortic retroperitoneal lymph node dissection should also be performed in order to decide whether further (systemic) adjuvant treatment is required and to provide an indication of prognosis. Based on the results of rapid histological analysis of the ovarian mass, known as ‘frozen section’, the surgeons will perioperatively decide whether or not to perform a full surgical staging procedure. Upon frozen section analysis, classification of ovarian neoplasms as borderline ovarian tumor or invasive carcinoma often is a real challenge, even for the well-trained pathologist. This is illustrated by the fact that 21% of borderline ovarian tumors diagnosed at frozen section examination turned out to be invasive cancer at the final pathology, for instance because there was a sampling error during frozen section analysis [4].

Another difficulty of intraoperative consultation in case of ovarian neoplasms may be the fact that it is not always possible for the pathologist to classify frozen section slides as a borderline ovarian tumor or an invasive carcinoma according to the World Health Organization (WHO) criteria [2]. In case of a borderline ovarian tumor showing equivocal or suspicious features for invasive carcinoma, an intermediate diagnosis, further denoted as “at least borderline”, is suggested [5]. In a recent systematic review and meta-analysis it was shown that a considerable number of women (25%) are diagnosed with “at least borderline” at frozen section and over 40% of these women are diagnosed with invasive carcinoma on paraffin section analysis. Full staging during initial surgery might be considered to prevent a second surgical procedure or chemotherapy in unstaged women, but is not clear whether this applies to all histologic subtypes [6]. It is important to note that the prevalence of positive lymph nodes is low in case of mucinous carcinoma with an expansile growth pattern, and a considerable number of surgeons do not perform a lymph node sampling in these cases [7–12]. Unfortunately, the systematic review did not have information about the number of serous vs mucinous subtypes of the “at least borderline” cases, and consequently also not about infiltrative vs expansile growth pattern in the case of a mucinous carcinoma. More detailed information about the histologic subtypes might help us with the dilemma of whether or not to perform a full ovarian cancer staging procedure. Therefore, the aim of this study was (i) to assess the prevalence of “at least borderline” frozen section results and (ii) to investigate the percentage of women having a final histological diagnosis of carcinoma following an “at least borderline” diagnosis at the time of frozen section examination, with a special interest in the histologic subtypes and growth patterns of the (invasive) tumors.

## Methods

The data of the present retrospective study was obtained from three hospitals in the Netherlands (Isala hospital, Zwolle; University Medical Centre Groningen, Groningen, and Maastricht University Medical Centre, Maastricht) after the Medical Ethical Review Committee approved conducting the study. All women who underwent ovarian surgery with perioperative frozen section analysis because of a suspicious adnexal mass in one of these hospitals from January 2007 to July 2018 were identified by searching the PALGA database (the Dutch nationwide histo- and cytopathology data network and archive) [13].

Women were included in case of a borderline ovarian tumor at frozen section and, at final pathology, a borderline ovarian tumor or invasive ovarian cancer. Women with a proven secondary tumor of the ovary (eg, metastasis of a gastrointestinal tumor) were excluded. Women’s baseline characteristics, the frozen section result and final histopathological diagnosis were retrieved from the patient files and saved into an encrypted database. Regarding the frozen section results, surgical procedure and pathology reports were carefully examined to verify whether the borderline frozen section result could or could not rule out invasive carcinoma. Women with a “rule out borderline” (maximum borderline ovarian tumor) or a decisive borderline ovarian tumor frozen section result (no suspicion of carcinoma) were reported as “borderline”, and women with a borderline ovarian tumor frozen section result in which the pathologist could not rule out invasive carcinoma were reported as “at least borderline”.

The data were analyzed with SPSS (IBM Corp. Released 2016. IBM SPSS Statistics for Windows, Version 25.0. Armonk, NY: IBM Corp.). The Fisher’s exact test and Fisher-Freeman-Halton test were executed to compare proportions of categorical outcomes according to different independent groups, and *p*-values < 0.05 were considered to indicate statistically significant differences.

## Results

In total, 223 women with a median age of 58 years (18–82) were included. Experienced gynaecopathologists are involved in the three participating hospitals. There was clear and extensive communication about the patients’ medical history and the intraoperative findings between gynecologist and pathologist at the time of frozen section. At least 2 samples and slides (dept. of Pathology, Maastricht University Medical Centre) and 3 samples and slides (dept. of Pathology, Isala Hospital, and dept. of Pathology, University Medical Center Groningen) were evaluated during frozen section irrespective of a straightforward diagnosis of a borderline tumor or an “at least borderline” tumor diagnosis.

Based on frozen section analysis, a straightforward diagnosis of a borderline tumor was observed in 182 of 223 women (81.6%) and an “at least borderline” tumor in 41 women (18.4%). When divided based on the histological subtype, 14 of 110 women (12.7%) with a serous tumor, 23 of 103 women (22.3%) with a mucinous tumor and 4 of 10 (40.0%) women with a tumor of a different histologic subtype were diagnosed with “at least borderline” at frozen section (*p* = 0.033, Fisher-Freeman-Halton test).

Final diagnosis, based on formalin fixated and paraffin embedded (FFPE) samples, revealed that 27 of these 223 women (12.1%) were diagnosed with invasive carcinoma as a final diagnosis. Of the 196 patients diagnosed with a borderline tumor, 171 (87.2%) had FIGO stage I disease and 25 (12.8%) were diagnosed with stage II disease. Twenty-one of twenty-seven patients (77.8%) with invasive carcinoma as a final diagnosis had FIGO stage I disease, 4 patients (14.8%) stage II and 2 patients (7.4%) stage III. Data on the gross examination of the tumor was available for all 27 upgraded tumors. Twenty of the upgraded tumors contain multiple solid and papillary areas, and most of these areas were sampled extensively. Irrespective of the frozen section result, 8 of 110 women (7.3%) with a serous tumor, 13 of 103 women (12.6%) with a mucinous tumor and 6 of 10 women (60.0%) with a tumor of a different histologic subtype were diagnosed with invasive carcinoma on paraffin section evaluation (*p* < 0.001, Fisher-Freeman-Halton Exact Test).

When distinguished by frozen section result, discordance was observed in 14 of 182 women (7.7%) with a borderline frozen section diagnoses and 13 of 41 women (31.7%) with an “at least borderline” frozen section diagnosis (*p* < 0.001, Fisher’s exact test) (Table 1). With respect to both straightforward borderline and “at least borderline” frozen section diagnoses, most carcinomas were diagnosed in the group of women with a histologic subtype of the tumor other than serous or mucinous (50.0 and 75.0%, respectively). Regarding the histologic subtypes, the proportion of women diagnosed with a carcinoma did only significantly differ from each other in case of straightforward borderline frozen section diagnoses (*p* = 0.001, Fisher-Freeman-Halton Exact Test) and not in case of “at least borderline” frozen section diagnoses (*p* = 0.088, Fisher-Freeman-Halton Exact Test). The increase in the risk of discordance was considered significant only in the case of serous tumors (*p* = 0.001, Fisher’s exact test) (Table 1).

**Table 1:** Concordance between diagnoses at frozen section examination and final paraffin diagnosis of 223 women with borderline ovarian tumor diagnoses

			Paraffin section diagnosis		
			Borderline	Carcinoma	*P*-value^*^
Frozen section diagnosis	Borderline (*n* = 182)		168 (92.3%)	14 (7.7%)	< 0.001
	At least borderline (*n* = 41)		28 (68.3%)	13 (31.7%)	
Frozen section diagnoses by histologic subtype	Serous (*n* = 110)	Borderline (*n* = 96)	93 (96.9%)	3 (3.1%)	0.001
		At least borderline (*n* = 14)	9 (64.3%)	5 (35.7%)	
	Mucinous (*n* = 103)	Borderline (*n* = 80)	72 (90.0%)	8 (10.0%)	0.129
		At least borderline (*n* = 23)	18 (78.3%)	5 (21.7%)	
	Other (*n* = 10)	Borderline (*n* = 6)	3 (50.0%)	3 (50.0%)	0.452
		At least borderline (*n* = 4)	1 (25.0%)	3 (75.0%)	

^*^Fisher’s Exact tests were performed

The group of women having tumors with a histologic subtype other than serous or mucinous consisted of 6 endometrioid tumors (3 “at least borderline” at frozen section), 2 Brenner tumors (both straightforward borderline at frozen section), 1 clear cell tumor (“at least borderline” at frozen section) and 1 seromucinous tumor (straightforward borderline at frozen section). Of the six women that showed invasive carcinoma as a final diagnosis, 5 women were diagnosed with an endometrioid carcinoma (3 preceded by an “at least borderline” frozen section diagnosis) and 1 woman with a malignant Brenner tumor (straightforward borderline frozen section). In total, five of 6 endometrioid tumors (83.3%) turned out to be invasive carcinoma on paraffin section evaluation.

Thirteen women were diagnosed with mucinous ovarian cancer, of which 5 showed the expansile growth pattern (38.5%) and 8 the infiltrative growth pattern (61.5%). Following a straightforward mucinous borderline frozen section (*n* = 80), 8 women (10.0%) were diagnosed with mucinous ovarian carcinoma diagnosis and 5 of these tumors (62.5%) showed an infiltrative growth pattern. Following an “at least borderline” frozen section diagnosis of mucinous histology (*n* = 23), 5 women (21.7%) were diagnosed with mucinous ovarian carcinoma, of which three (60.0%) had shown an infiltrative growth pattern. There was no relation between frozen section result and growth pattern in case of mucinous ovarian carcinoma (*p* = 1.000).

## Discussion

On a regular basis, it is hard for the pathologist to report a frozen section diagnosis as a borderline ovarian tumor or an invasive carcinoma according to the WHO criteria and, sometimes, “at least borderline” is used [2, 5]. It is of value to be aware of the number of women with an “at least borderline” diagnosis that will have carcinoma as the final diagnosis, as this might have implication for clinical practice. After conducting a systematic review and meta-analysis regarding this subject, here, we performed a retrospective cohort study that evaluated more than 200 women with borderline and “at least borderline” frozen section diagnoses with a special interest in the histologic subtype of the tumors [6].

First of all, we showed that approximately 20% of borderline ovarian tumor frozen section diagnoses are reported as “at least borderline” because of features that are suspicious but not convincing enough to speak of invasive carcinoma. Of these women, permanent histology evaluation shows invasive carcinoma in an average of 30% of women, but up to 35.7% for serous tumors and 75% of tumors with a histologic subtype other than serous or mucinous (mostly endometrioid tumors). Subclassification of all borderline ovarian tumor frozen section diagnoses into straightforward borderline and “at least borderline” frozen section diagnoses seems to reduce the discordance rate of (straightforward) borderline frozen section diagnoses when compared to the literature (21% vs 7.7%) [4]. When subdivided by histologic subtype, the increased proportion of women diagnosed with a carcinoma as final diagnosis following a diagnosis of “at least borderline” at frozen section was only significant for women with a serous tumor in our retrospective analysis.

Because of the considerable and increased chance of a final diagnosis of carcinoma following a frozen section diagnosis of “at least borderline”, especially in the serous subtype, full surgical staging at the initial surgery in these cases might be considered. Irrespective of the frozen section result (straightforward borderline or “at least borderline”), all but one (5 of 6) endometrioid tumors were diagnosed with invasive carcinoma on paraffin section analysis. However, the number of cases is too low for drawing definitive conclusions. In cases where final diagnosis shows invasive cancer, incomplete staging and the subsequent indication for adjuvant chemotherapy or a second surgical staging procedure, with all its potential consequences, may be avoided by this strategy [7, 14]. However, women, in whom full ovarian cancer staging is performed and the final diagnosis shows a borderline ovarian tumor, are exposed to the risks of surgical over-treatment, which might lead to several peri- and postoperative risks such as lymphocysts or lymphedema following a lymph node sampling. As part of shared decision making the aforementioned potential risks and benefits of performing additional staging procedures at the time of initial surgery should be discussed with the woman at the outpatient clinic. Of course, other factors may influence the decision to perform a full surgical staging procedure at the time of the initial surgery, such as patient characteristics (eg, age or wish for fertility-sparing surgery) and other factors such as macroscopic appearance of the tumor and preoperative CA-125 levels [1, 15].

There may be some additional reasons to question whether one should perform full staging in cases of a frozen section diagnosis of an “at least borderline” ovarian tumor with a mucinous histologic subtype. The prevalence of women with positive lymph nodes is low in cases of suspected FIGO stage I mucinous carcinoma with an expansile growth pattern (0.9–2.6%). Therefore, some surgeons do not perform a lymph node sampling in these tumors with an expansile growth pattern [7–12]. In the present study, the risk of carcinoma as final histological diagnosis was not significantly higher following a mucinous “at least borderline” frozen section diagnosis, when compared to a straightforward borderline frozen section diagnosis (10.0 vs 21.7%). Furthermore, almost half tumors showed the expansile growth pattern and the distribution did not differ between the straightforward borderline and “at least borderline” frozen section results. Thus, for these reasons, one may question whether full surgical staging is necessary at the time of the initial surgery when frozen section evaluation shows a mucinous borderline tumor with features suspected of mucinous carcinoma. The present study has some limitations. First of all, the study has a retrospective design, so we had to deal with missing data. Furthermore, because borderline ovarian tumors are rare and the study aimed at a specific group of women with a borderline tumor (including rule out borderline) or “at least borderline” frozen section diagnosis, the number of women included in the retrospective analysis and subsequent subgroups was relatively low. Our recent systematic review and meta-analysis confirmed the results of our retrospective study, indicating that the results in our study might be considered as robust [6]. However, a considerable issue might be the fact that we did not include the women with a cystadenoma as a final histological diagnosis, which means that percentages of women with a borderline ovarian tumor or invasive carcinoma as a final diagnosis might be slightly overrated. Overall, around 6 % of women with a borderline frozen section diagnosis show a benign cystadenoma on paraffin section evaluation. Assuming that this percentage is lower with respect to “at least borderline” frozen section diagnoses, the difference between proportion of women diagnosed with an invasive carcinoma following straightforward borderline and “at least borderline” frozen section diagnoses will be increased [4]. A good frozen section diagnosis relies on (i) clear and extensive communication about the patients’ history between gynecologist and pathologist, (ii) thoroughly gross examination by the pathologist and (iii) extensive sampling. In the three participating hospitals 2 or 3 samples/slides were evaluated during frozen section with clear and extensive communication at the time of frozen section. Furthermore, thoroughly gross examination was performed by the pathologist.

However, additional sampling in case of an “at least borderline” diagnosis was not performed routinely. Additional sampling might avoid unnecessary full staging surgery, and may indeed prolong the time of intraoperative consultation. The prevalence of “at least borderline” tumor diagnosis in our study was similar as found in the recent systematic review and meta-analysis [6]. In the three participating hospitals, diagnosis of “at least borderline” was based on 2 or 3 samples (see above). No additional sampling has been performed, so undersampling cannot be ruled out. It may be important to further evaluate this, as the findings of the current study and the systematic review and meta-analysis suggest that this may be an important issue.

## Conclusions

In conclusion, this study shows that the overall risk of carcinoma following an “at least borderline” frozen section diagnosis is comparable to a recent review and systematic analysis (31.7%), but also that it varies by histologic subtype (21.7, 35.7 and 75.0% for mucinous, serous and other histologic tumors, respectively). Especially in case of serous “at least borderline” frozen section results and endometrioid frozen section diagnoses, there was an increased risk of finding invasive carcinoma as final diagnosis. Furthermore, a considerable number of cases of invasive mucinous carcinomas showed the expansile growth pattern, which have a low change of positive lymph nodes. Thus, full staging at the time of initial surgery might be considered in order to prevent a second procedure for the serous histologic subtype. However, future studies are needed to evaluate whether improvement of sampling protocols (additional sampling in case of an “at least borderline” diagnosis) during frozen section examination, as well as finding more differentiating criteria what will lead to specific training of pathologists with respect to discrimination of these tumor categories, could improve the reporting of frozen section diagnostics.

## Data Availability

The datasets used and/or analysed during the current study are available from the corresponding author on reasonable request.
